# Supplementary material for: Long-Term Impact of COVID-19 on Mental Health among the General Public: A Nationwide Longitudinal Study in China
Source: Int J Environ Res Public Health. 2021 Aug 20;18(16):8790. doi: 10.3390/ijerph18168790 (PMC8393580; doi:10.3390/ijerph18168790)

Supplementary Online Content

Long-term impact of COVID-19 on mental health among the general public: a nationwide longitudinal study in China

Table S1. STROBE checklist for cohort studies.

Table S2. Summary of questions of the questionnaire related to this study.

Table S3. Factors associated with long-term mental health symptoms in the multivariable regression model.

Table S4. Changes in prevalence of mental health symptoms from baseline to follow-up stratified by demographic and epidemic-related factors.

Figure S1. Changes in scores of depression, anxiety and insomnia from baseline to follow-up stratified by COVID-19 resurgence

Figure S2. Changes in scores of depression, anxiety and insomnia from baseline to follow-up stratified by increases in work burden after resuming work.

Figure S3. Changes in scores of depression, anxiety and insomnia from baseline to follow-up stratified by educational level.

Figure S4. Changes in scores of depression, anxiety and insomnia from baseline to follow-up stratified by voluntarily wearing face masks.

Figure S5. Changes in scores of depression, anxiety and insomnia from baseline to follow-up stratified by voluntarily reducing social gatherings.

Figure S6. Changes in scores of depression, anxiety and insomnia from baseline to follow-up stratified by seeking psychological consultation.

Table S1- STROBE checklist for cohort studies

	Item No	Recommendation	Section/ Paragraph
**Title and abstract**	1	(*a*) Indicate the study's design with a commonly used term in the title or the abstract	Title & Abstract
		(*b*) Provide in the abstract an informative and balanced summary of what was done and what was found	Author Summary & Abstract
**Introduction**			
Background/rationale	2	Explain the scientific background and rationale for the investigation being reported	Introduction paragraph 1-2
Objectives	3	State specific objectives, including any prespecified hypotheses	Introduction paragraph 3
**Methods**			
Study design	4	Present key elements of study design early in the paper	Methods-Study design
Setting	5	Describe the setting, locations, and relevant dates, including periods of recruitment, exposure, follow-up, and data collection	Methods-Study design
Participants	6	(*a*) Give the eligibility criteria, and the sources and methods of selection of participants. Describe methods of follow-up	Methods-Participants and Fig 1
		(*b*) For matched studies, give matching criteria and number of exposed and unexposed	-
Variables	7	Clearly define all outcomes, exposures, predictors, potential confounders, and effect modifiers. Give diagnostic criteria, if applicable	Methods-Measures and variables
Data sources/ measurement	8*	For each variable of interest, give sources of data and details of methods of assessment (measurement). Describe	Methods-Measures and variables

		comparability of assessment methods if there is more than one group	
Bias	9	Describe any efforts to address potential sources of bias	Methods-Statistical analyses paragraph 1
Study size	10	Explain how the study size was arrived at	Methods-Participants
Quantitative variables	11	Explain how quantitative variables were handled in the analyses. If applicable, describe which groupings were chosen and why	Methods-Measures and variables paragraph 1
Statistical methods	12	(*a*) Describe all statistical methods, including those used to control for confounding	Methods-Statistical analyses
		(*b*) Describe any methods used to examine subgroups and interactions	Methods-Statistical analyses paragraph 2
		(*c*) Explain how missing data were addressed	-
		(*d*) If applicable, explain how loss to follow-up was addressed	Methods-Statistical analyses paragraph 1
		(*e*) Describe any sensitivity analyses	-
**Results**			
Participants	13*	(a) Report numbers of individuals at each stage of study—eg numbers potentially eligible, examined for eligibility, confirmed eligible, included in the study, completing follow-up, and analysed	Methods-Participants, Fig 1 and Table 1
		(b) Give reasons for non-participation at each stage	Methods-Participants and Fig 1
		(c) Consider use of a flow diagram	Fig 1
Descriptive data	14*	(a) Give characteristics of study participants (eg demographic, clinical, social) and information on exposures and potential confounders	Results- Demographic and epidemic-related characteristics of participants
		(b) Indicate number of participants with missing data for each variable of interest	Table 1

		(c) Summarise follow-up time (eg, average and total amount)	Abstract, Author summary, Methods-Study design
Outcome data	15*	Report numbers of outcome events or summary measures over time	Table 2, Table 3 and S3 Table
Main results	16	(*a*) Give unadjusted estimates and, if applicable, confounder-adjusted estimates and their precision (eg, 95% confidence interval). Make clear which confounders were adjusted for and why they were included	Table 2, Table 3 and S3 Table
		(*b*) Report category boundaries when continuous variables were categorized	Methods – Measures and variables paragraph 1
		(*c*) If relevant, consider translating estimates of relative risk into absolute risk for a meaningful time period	Table 5 and S1-6 Figure
Other analyses	17	Report other analyses done—eg analyses of subgroups and interactions, and sensitivity analyses	Fig 2, S3 Table and S1-6 Figure
**Discussion**			
Key results	18	Summarise key results with reference to study objectives	Discussion paragraph 1 and Conclusions
Limitations	19	Discuss limitations of the study, taking into account sources of potential bias or imprecision. Discuss both direction and magnitude of any potential bias	Discussion paragraph 9
Interpretation	20	Give a cautious overall interpretation of results considering objectives, limitations, multiplicity of analyses, results from similar studies, and other relevant evidence	Discussion paragraph 2-9
Generalisability	21	Discuss the generalisability (external validity) of the study results	Discussion paragraph 9
**Other information**			

Funding	22	Give the source of funding and the role of the funders for the present study and, if applicable, for the original study on which the present article is based	Funding

*Give information separately for exposed and unexposed groups.

Note: An Explanation and Elaboration article discusses each checklist item and gives methodological background and published examples of transparent reporting. The STROBE checklist is best used in conjunction with this article (freely available on the Web sites of PLoS Medicine at http://www.plosmedicine.org/, Annals of Internal Medicine at http://www.annals.org/, and Epidemiology at http://www.epidem.com/). Information on the STROBE Initiative is available at http://www.strobe-statement.org.

Table S2 - Summary of questions of the questionnaire related to this study.

Questions	Types	Choices
**Part One: Demographic information**		
**Please select your gender:**	Single choice	Male/Female
**Please fill in your age (years):**	Blank	/
**Please fill in your current living geographical region:**	Blank	/
**Your current living area is:**	Single choice	Urban/Rural
**Please fill in your permanent geographical region:**	Blank	/
**Please select your highest level of education:**	Single choice	Elementary school or below/Junior high school/Senior high school or vocational school/Bachelor degree or college diploma/Master degree or above
**Please select your current marital status:**	Single choice	Married/Unmarried
**Please select your monthly family income (yuan):**	Single choice	<1000/1000-2999/3000-4999/5000-7999/8000-11999/≥12000
**Do you have a history of chronic diseases (hypertension, diabetes, cerebrovascular disease, heart diseases, and malignant tumors)?**	Single choice	Yes/Unknown/No
**Do you have a history of psychiatric disorders?**	Single choice	Yes/Unknown/No
**Please select the type of psychiatric disorder you suffer from: (this depends on "Yes" being checked on the last question)**	Multiple choice	Depression/Bipolar disorder/ Schizophrenia/Anxiety/Obsessive-compulsive disorder/Attention deficit hyperactivity disorder/Others/Unknown
**Do you have a family history of psychiatric disorders?**	Single choice	Yes/Unknown/No
**Have you ever had any sleep problem?**	Single choice	Yes/No
**Do you have any habit of smoking?**	Single choice	Yes/No
**Do you have any habit of drinking alcohol?**	Single choice	Yes/No
**Please select your current occupation:**	Single choice	Ordinary full-time students/Medical students/ Graduates/Medical staff/Other medical

		workers/Scientific researchers/The police, the security guards/Workers in the transportation industry/Community workers or social workers/Other administrative staff/Clerical staff/Other agricultural personnel/Other commercial or service staff/Other professionals (e.g. accountants, lawyers, architects, journalists, etc.)/Other workers/Military personnel/Self-employment or freelancers/Unemployed/Others
**Part Two: Epidemic-related questions**		
**Are you infected with COVID-19?**	Single choice	Confirmed cases/Suspected cases/Not infected
**Have any of your family members or friends been infected with COVID-19?**	Single choice	Yes/No
**Have you come in close contact with patients infected with COVID-19?**	Single choice	Yes/No
**Have you been to Hubei province in the past 2 months?**	Single choice	Yes/No
**Please comment on your level of concern about the COVID-19 epidemic:**	Enter number from 0 (very unconcerned) to 10 (very concerned)	/
**Please select the main channels for you to obtain information about the COVID-19 epidemic?**	Multiple choice	TV news/Network news/Search engines (e.g. Google, etc.)/Social platform (e.g. WeChat, chat group, ins, etc.)/Broadcast/Paper media/Newspaper brochure/Others
**Please comment on your level of understanding of the COVID-19 epidemic:**	Enter number from 0 (understand very little) to 10 (understand very well)	/
**Are you a frontline worker of COVID-19?**	Single choice	Yes/No
**Are any of your family members or friends frontline workers?**	Single choice	Yes/No

**Are you back to work now?**	Single choice	Yes/No
**If you are currently working, what is your workplace? (this depends on "Yes" being checked in the last question)**	Single choice	Work at home/Work not at home
**Are you likely to be exposed to other people at work?**	Single choice	Close contact (≤1.5 m) to patients infected with COVID-19/Non-close contact (＞1.5 m) with patients infected with COVID-19/Close contact (≤1.5 m) with patients with other diseases/Non-close contact (＞1.5 m) with patients with other diseases/Close contact (≤1.5 m) with general people/Non-close contact (＞1.5 m) with general people/Not at work, work at home, or without exposure to people at work
**What is the impact of the COVID-19 pandemic on your work?**	Multiple choice	Unemployment/Postponement of work/Impact on income/ Busier than ever/No impact/Others
**Part Three: Isolation conditions and social attitudes toward the COVID-19 pandemic**		
**Was there any traffic control in your area during the pandemic?**	Single choice	Yes/No
**Do you live in a community that restricts people's access?**	Single choice	Yes/No
**Have you ever experienced quarantine?**	Single choice	Centralized quarantine/Home quarantine/No quarantine
**How long have you been quarantining? (this depends on "Yes" being checked on the last question)**	Single choice	Fewer than 7 days/7-13 days/14 days/More than 14 days
**Do you quarantine as required? (this depends on "Yes" being checked on the last question)**	Single choice	Yes/No
**If you don’t quarantine as required, what’s the reason? (this depends on "No" being checked on the last question)**	Multiple choice	I must go out under special circumstances/I don’t need quarantine since I’m not infected /I cannot stand the feeling of boringness and loneliness due to quarantine/Others

**To what extent are you worried about being infected with COVID-19?**	Single choice	Not at all/Slight/Moderate/Significant/Severe
**How long do you think the pandemic will take to be controlled?**	Single choice	Fewer than 1 month/2-3 months/4-6 months/More than 5 months
**What do you think are the difficulties in the current pandemic prevention and control?**	Multiple choice	Shortage of protection materials/Insufficient medical personnel and medical resources/Patients/People under quarantine don't understand or cooperate/People are not fully aware of the importance of self-protection/Mental problems/Others
**Are you stressed due to the local pandemic and various restriction policy?**	Single choice	Yes/No
**What's your main stressor?(this depends on "Yes" being checked on the last question)**	Multiple choice	The severity of the pandemic/Various access restriction policies/Worries about their own and family members' safety/Worries about the safety of medical personnel working in frontline/Others
**To what extent you feel stressed before the pandemic?**	Enter the number from 0 (no stress) to 10 (extreme stress)	/
**Would you like to learn psychological knowledge disseminated by media before the pandemic?**	Single choice	Yes/No
**Would you like to learn psychological intervention and therapy knowledge before the pandemic?**	Single choice	Yes/No
**To what extent the difficulty you encountered in finding information about psychological therapy and intervention before pandemic?**	Enter the number from 0 (not difficult at all) to 10 (extremely difficult)	/
**To what extent you feel stressed after the pandemic?**	Enter the number from 0 (not stressed	/

	at all) to 10 (extremely stressed)	
**Would you like to learn psychological knowledge disseminated by media after the pandemic?**	Single choice	Yes/No
**Would you like to learn psychological intervention and therapy knowledge after the pandemic?**	Single choice	Yes/No
**To what extent the difficulty you encountered in finding information about psychological therapy and intervention after pandemic?**	Enter the number from 0 (not difficult at all) to 10 (extremely difficult)	/
**I would report and seek treatment if I was infected**	Single choice	Yes/No
**For what reason you would not report and seek treatment? (this depends on "No" being checked on the last question)**	Multiple choice	Worries about the judgement of people/Worries about being labeled/Impact on later life/Invasion of privacy/Family factors (e.g. worries about parents or children, etc.)/Worries about treatment effects/Others
**Are you in Hubei now?**	Single choice	Yes, I'm in Wuhan/Yes, I'm in another city of Hubei/No
**Did you return from Hubei to your current location?(this depends on "No" being checked on the last question)**	Single choice	Yes, I returned from Wuhan /Yes, I returned from another city of Hubei/No
**Social attitudes towards and perceived discrimination among people in the most severely affected area by COVID-19**	Items and scales	/
**Part Four: Questions specific to the follow-up survey inquiring about conditions after initial COVID-19 peak**		
**Have you experienced COVID-19 resurgence (reemergence of new COVID-19 cases after the report of zero new cases) in your living place?**	Single choice	Yes/No

**Do you perceive increases in work burden after resuming work?**	Single choice	Yes/No
**Do you voluntarily wear face masks when going out now?**	Single choice	Yes/No
**Do you voluntarily reduce gatherings now?**	Single choice	Yes/No
**Have you sought psychological consultation since the outbreak?**	Single choice	Yes/No

Table S3-Factors associated with long-term mental health symptoms in the multivariable regression model.

Factors	Definitions	Types of variables	Reference category	Summarizing forms
**Demographic factors**				
**Gender**	In the following 2 categories: 1.male; 2.female	Categorical	Female	Odds ratio with a 95% confidence interval
**Age(years)**	Age	Continuous	/	Odds ratio with a 95% confidence interval
**Living area**	In the following 2 categories:1.urban; 2.rural	Categorical	Rural	Odds ratio with a 95% confidence interval
**Educational level**	In the following 2 categories: 1. College school or higher; 2. lower than college school	Categorical	<college	Odds ratio with a 95% confidence interval
**Marital status**	In the following 2 categories: 1.married; 2.unmarried	Categorical	Unmarried	Odds ratio with a 95% confidence interval
**Monthly family income (yuan)**	In the following 2 categories: 1.<5000; 2. ≥ 5000	Categorical	≥ 5000	Odds ratio with a 95% confidence interval
**History of chronic diseases**	In the following 2 categories:1.yes (if the participant had a history of chronic diseases, including hypertension, diabetes, cardiovascular diseases, heart diseases and malignant tumors); 2.unknown (if the participant reported to be unclear of their own history of psychiatric disorders) or no (otherwise)	Categorical	Unknown or no	Odds ratio with a 95% confidence interval
**History of psychiatric disorders**	In the following 2 categories:1.yes (if the participant had a history of psychiatric disorders); 2.unknown (if the participant reported to be unclear of their own history of psychiatric disorders) or no (otherwise)	Categorical	Unknown or no	Odds ratio with a 95% confidence interval
**Family history of psychiatric disorders**	In the following 2 categories: 1.yes (if the participant had a family history of psychiatric disorders); 2.unknown (if the participant reported to be unclear of their family history of psychiatric disorders) or no (otherwise)	Categorical	Unknown or no	Odds ratio with a 95% confidence interval
**Personal factors associated with COVID-19**				
**Confirmed or suspected cases of COVID-19**	In the following 2 categories:1.yes (if the participant reported to be confirmed or suspected cases in either baseline or follow-up survey); 2.no otherwise	Categorical	No	Odds ratio with a 95% confidence interval

**Family members of COVID-19 patients**	In the following 2 categories: 1.yes (if the participant reported to have family members infected with COVID-19 in baseline or follow-up survey);2. no otherwise	Categorical	No	Odds ratio with a 95% confidence interval
**Living in province most severely affected by initial outbreak**	In the following 2 categories: 1.yes (if the participant reported to live in province most severely affected by initial outbreak in baseline or follow-up survey); 2.no otherwise	Categorical	No	Odds ratio with a 95% confidence interval
**Living in places with COVID-19 resurgences (collected in follow-up survey)**	In the following 2 categories: 1. yes (if the participant reported to live in places with COVID-19 resurgences in follow-up survey);2.no otherwise	Categorical	No	Odds ratio with a 95% confidence interval
**Experiences of quarantine**	In the following 2 categories: 1.yes (if the participant reported to have quarantine experiences in either baseline or follow-up study); 2.no otherwise	Categorical	No	Odds ratio with a 95% confidence interval
**Wearing face masks voluntarily when going out (collected in follow-up survey)**	In the following 2 categories: 1.yes (if the participant reported to voluntarily wear masks in public places in follow-up survey); 2. no otherwise	Categorical	No	Odds ratio with a 95% confidence interval
**Reducing gatherings voluntarily (collected in follow-up survey)**	In the following 2 categories: 1.yes (if the participant reported to voluntarily reduce gatherings in follow-up survey); 2. no otherwise	Categorical	No	Odds ratio with a 95% confidence interval
**Seeking psychological consultation since the COVID-19 outbreak (collected in follow-up survey)**	In the following 2 categories: 1.yes (if the participant reported to have sought psychological consultation since COVID-19 outbreak in follow-up survey);2. no otherwise	Categorical	No	Odds ratio with a 95% confidence interval
**Work-related factors associated with COVID-19**				
**Direct engagement in work related to COVID-19 control**	In the following 2 categories: 1.yes (if the participant reported to directly participate in the control of COVID-19, covering a wide range of occupations including medicine, research, public health, media,	Categorical	No	Odds ratio with a 95% confidence interval

	security work, police, community work, emergency material delivery services, charity, construction, management, and psychological interventions) in either baseline or follow-up survey); 2.no otherwise			
**Self-perceived occupational exposure risk to COVID-19**	In the following 2 categories: 1.yes (if the participant reported to be have occupational exposure risk to COVID-19 in either baseline or follow-up survey);2. no otherwise	Categorical	No	Odds ratio with a 95% confidence interval
**Self-perceived increases in work burden after resuming work (collected in follow-up survey)**	In the following 2 categories: 1.yes (if the participant reported self-perceived increases in work burden compared with before the outbreak in follow-up survey; 2. no otherwise	Categorical	No	Odds ratio with a 95% confidence interval

Table S4- Changes in prevalence of mental health symptoms from baseline to follow-up stratified by demographic and epidemic-related factors.

Factors	Any mental health symptoms		Depression		Anxiety		Insomnia	
	n (weighted %, 95%CI) at baseline survey	n (weighted %, 95%CI) at follow-up survey	n (weighted %) at baseline survey	n (weighted %) at follow-up survey	n (weighted %) at baseline survey	n (weighted %) at follow-up survey	n (weighted %) at baseline survey	n (weighted %) at follow-up survey
**Gender**								
Male (n=5026)	2442 (48.6) [47.2-50.0]	2447 (48.7) [47.3-50.1]	1687 (33.6)	1888 (37.6)*	1838 (36.6)	1796 (35.7)	1684 (33.5)	1958 (39.0)*
Female (n=5466)	2423 (44.3) [43.0-45.7]	2287 (41.8) [40.5-43.2]*	1464 (26.8)	1640 (30.0)*	1854 (33.9)	1619 (29.6)*	1443 (26.4)	1743 (31.9)*
**Age**								
18-39 (n=7306)	3486 (47.7) [46.6-48.9]	3481 (47.7) [46.5-48.8]	2351 (32.2)	2661 (36.4)*	2704 (37.0)	2587 (35.4)*	2209 (30.2)	2714 (37.1)*
≥40 (n=3186)	1379 (43.3) [41.6-45.0]	1252 (39.3) [37.6-41.0]*	800 (25.1)	867 (27.2)*	989 (31.0)	828 (26.0)*	918 (28.8)	987 (31.0)*
**Living area**								
Urban (n=9781)	4506 (46.1) [45.1-47.1]	4389 (44.9) [43.9-45.9]*	2910 (29.8)	3254 (33.3)*	3418 (34.9)	3138 (32.1)*	2901 (29.7)	3427 (35.0)*
Rural (n=711)	359 (50.5) [46.7-54.2]	345 (48.5) [44.8-52.2]	241 (33.9)	274 (38.5)*	275 (38.7)	277 (38.9)	226 (31.8)	274 (38.5)*
**Educational level**								
College school or higher (n=8726)	4042 (46.3) [45.3-47.4]	4009 (45.9) [44.9-47.0]	2615 (30.0)	2976 (34.1)*	3056 (35.0)	2872 (32.9)*	2624 (30.1)	3136 (35.9)*
Lower than college school (n=1766)	823 (46.6) [44.3-49.0]	725 (41.0) [38.7-43.4]*	536 (30.3)	552 (31.2)	637 (36.1)	543 (30.7)*	503 (28.5)	565 (32.0)*
**Marital status**								
Married (n=8101)	3651 (45.1) [44-46.2]	3465 (42.8) [41.7-43.9]*	2313 (28.5)	2555 (31.5)*	2802 (34.6)	2493 (30.8)*	2295 (28.3)	2691 (33.2)*
Unmarried (n=2391)	1214 (50.8) [48.7-52.8]	1268 (53.0) [51.0-55.1]*	838 (35.1)	973 (40.7)*	891 (37.3)	922 (38.6)	832 (34.8)	1010 (42.2)*
**Family income level(yuan)**								
<5000 (n=2402)	1173 (48.8) [46.8-50.9]	1157 (48.2) [46.1-50.2]	807 (33.6)	885 (36.9)*	900 (37.5)	873 (36.3)	725 (30.2)	909 (37.8)*
≥5000 (n=8090)	3692 (45.6) [44.5-46.7]	3577 (44.2) [43.1-45.3]*	2344 (29.0)	2642 (32.7)*	2793 (34.5)	2542 (31.4)*	2402 (29.7)	2792 (34.5)*
**Family members of COVID-19 patients**								
Yes (n=221)	164 (74.3) [67.9-79.8]	158 (71.8) [65.3-77.5]	123 (55.5)	133 (60.3)	140 (63.3)	123 (55.5)	126 (57.3)	128 (58.0)
No (n=10271)	4701 (45.8) [44.8-46.7]	4575 (44.5) [43.6-45.5]*	3028 (29.5)	3394 (33.0)*	3553 (34.6)	3292 (32.1)*	3001 (29.2)	3573 (34.8)*

**Living in provinces most severely affected by initial peak**								
Yes (n=623)	390 (62.5) [58.5-66.3]	361 (57.9) [53.9-61.8]*	291 (46.6)	301 (48.3)	326 (52.3)	296 (47.5)*	273 (43.9)	297 (47.6)
No (n=9869)	4476 (45.4) [44.4-46.3]	4372 (44.3) [43.3-45.3]	2860 (29.0)	3227 (32.7)*	3367 (34.1)	3119 (31.6)*	2854 (28.9)	3404 (34.5)*
**Quarantine**								
Yes (n=3752)	1971 (52.5) [50.9-54.1]	1976 (52.7) [51.1-54.3]	1379 (36.8)	1546 (41.2)*	1555 (41.5)	1518 (40.5)	1304 (34.8)	1568 (41.8)*
No (n=6740)	2894 (42.9) [41.8-44.1]	2758 (40.9) [39.7-42.1]*	1772 (26.3)	1981 (29.4)*	2138 (31.7)	1897 (28.1)*	1823 (27.0)	2133 (31.6)*
**Occupational exposure risk to COVID-19**								
Yes (n=1430)	804 (56.2) [53.6-58.8]	799 (55.9) [53.3-58.5]	590 (41.3)	650 (45.5)*	682 (47.7)	638 (44.6)*	538 (37.7)	631 (44.1)*
No (n=9062)	4061 (44.8) [43.8-45.8]	3935 (43.4) [42.4-44.4]*	2561 (28.3)	2878 (31.8)*	3011 (33.2)	2777 (30.6)*	2589 (28.6)	3070 (33.9)*
**Living in places with resurgences**								
Yes (n=2060)	1062 (51.6) [49.4-53.8]	1109 (53.8) [51.7-56.0]	702 (34.1)	836 (40.6)*	789 (38.3)	812 (39.4)	730 (35.4)	874 (42.4)*
No (n=8432)	3803 (45.1) [44.0-46.2]	3624 (43.0) [41.9-44.0]*	2449 (29.0)	2692 (31.9)*	2904 (34.4)	2602 (30.9)*	2397 (28.4)	2827 (33.5)*
**Self-perceived increases in work burden after resuming work**								
Yes (n=4457)	2415 (54.2) [52.7-55.6]	2472 (55.5) [54.0-56.9]	1640 (36.8)	1928 (43.3)*	1870 (42.0)	1890 (42.4)	1621 (36.4)	1970 (44.2)*
No (n=6035)	2450 (40.6) [39.4-41.9]	2262 (37.5) [36.3-38.7]*	1510 (25.0)	1600 (26.5)*	1823 (30.2)	1525 (25.3)*	1506 (25.0)	1731 (28.7)*
**Wearing face masks voluntarily when going out**								
Yes (n=10091)	4645 (46.0) [45.1-47.0]	4492 (44.5) [43.5-45.5]*	2980 (29.5)	3327 (33.0)*	3518 (34.9)	3215 (31.9)*	2976 (29.5)	3495 (34.6)*
No (n=401)	220 (54.8) [49.7-59.7]	241 (60.1) [55.1-64.9]	170 (42.5)	201 (50.0)*	175 (43.6)	200 (49.8)*	151 (37.7)	206 (51.4)*
**Seeking psychological consultation since the COVID-19 outbreak**								
Yes (n=1641)	1070 (65.2) [62.8-67.5]	1191 (72.6) [70.3-74.7]*	837 (51.0)	1045 (63.7)*	894 (54.5)	1040 (63.3)*	758 (46.2)	1003 (61.1)*
No (n=8851)	3795 (42.9) [41.8-43.9]	3542 (40.0) [39.0-41.1]*	2313 (26.1)	2482 (28.0)*	2799 (31.6)	2375 (26.8)*	2369 (26.8)	2697 (30.5)*
**Reducing social gatherings since COVID-19 outbreak**								
Yes (n =9340)	4290 (45.9) [44.9-46.9]	4128 (44.2) [43.2-45.2]*	2718 (29.1)	3042 (32.6)*	3246 (34.7)	2946 (31.5)*	2724 (29.2)	3191 (34.2)*
No (n=1152)	575 (49.9) [47.0-52.9]	605 (52.6) [49.6-55.5]	433 (37.6)	485 (42.1)*	447 (38.8)	469 (40.7)	403 (35.0)	510 (44.3)*

Depression is defined as having a Patient Health Questionnaire-9 score≥5. Anxiety is defined as having a Generalize Anxiety Disorder-7 score ≥5. Insomnia is defined as having an Insomnia Severity Index score ≥8. Any mental health symptoms is defined as having either depression, anxiety and insomnia. * P<0·05 for McNemar tests for statistically significant differences in prevalence of mental health symptoms in baseline vs follow-up survey.

Figure S1- Changes in scores of depression, anxiety and insomnia from baseline to follow-up stratified by COVID-19 resurgences. Values are estimated marginal means from multivariable generalized linear mixed models. *P* values are for factor by time interaction terms. The error bars indicate 95%CIs.

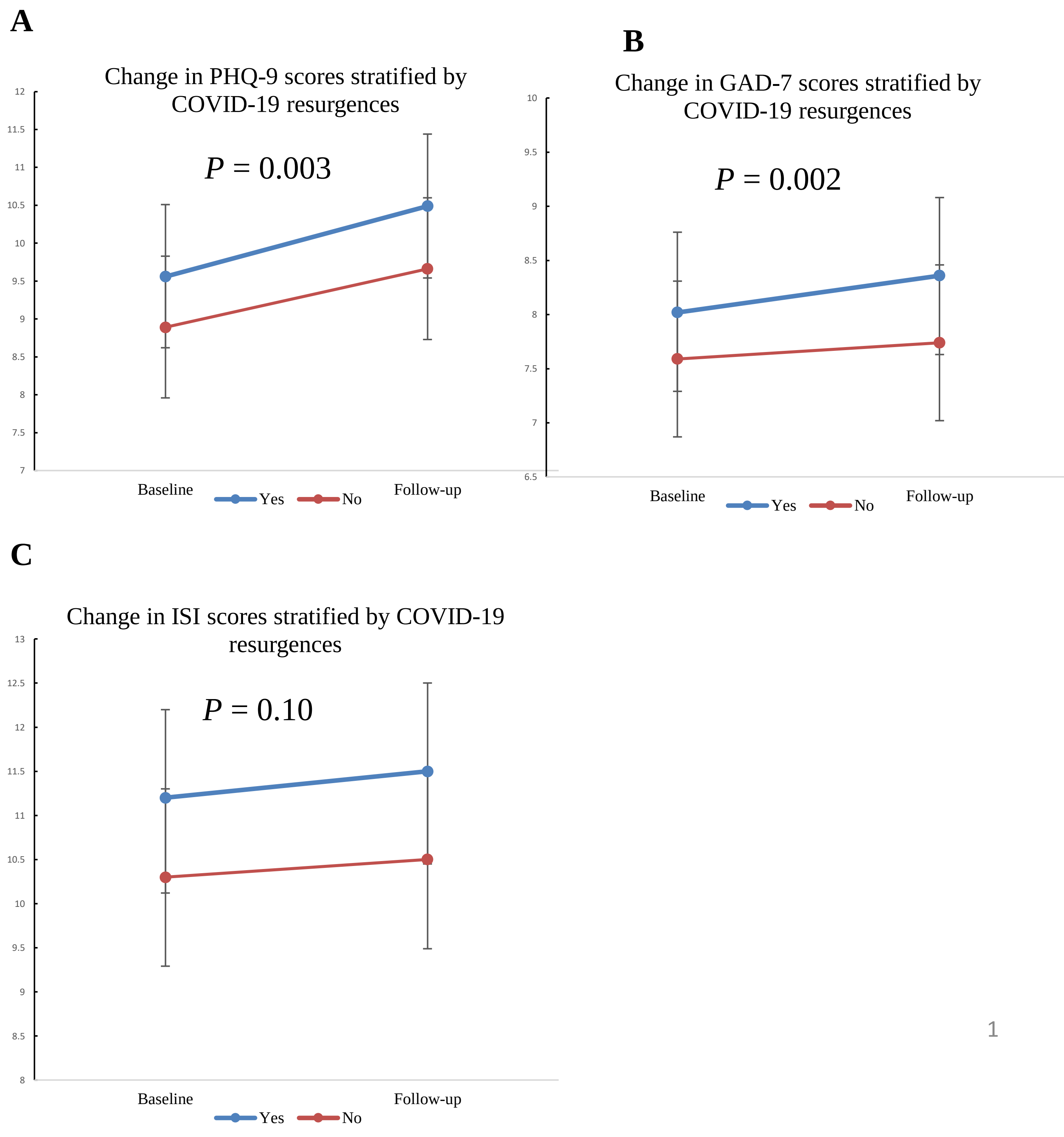

Figure S2- Changes in scores of depression, anxiety and insomnia from baseline to follow-up stratified by increases in work burden after resuming work. Values are estimated marginal means from multivariable generalized linear mixed models. *P* values are for factor by time interaction terms. The error bars indicate 95%CIs.

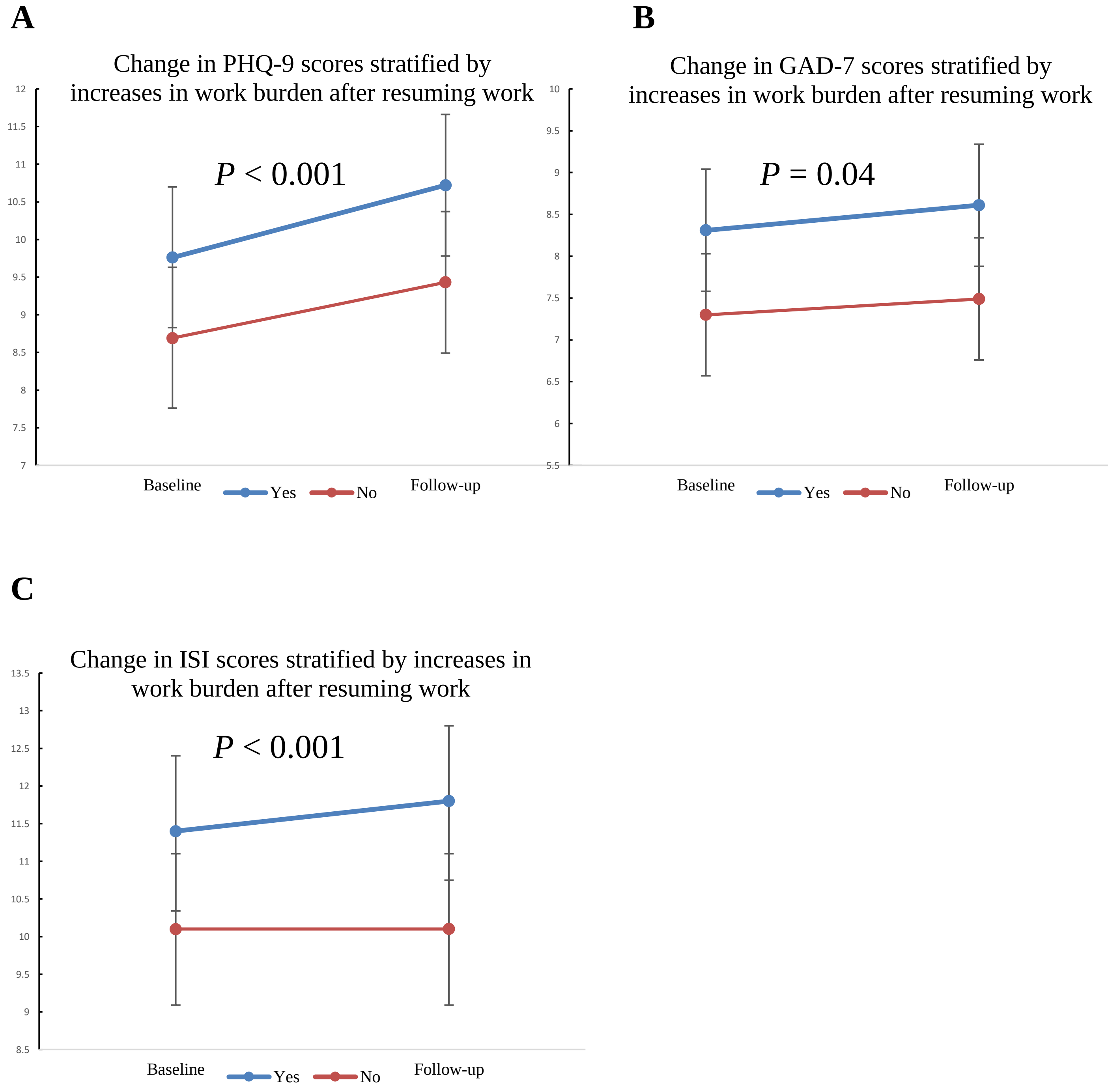

Figure S3- Changes in scores of depression, anxiety and insomnia from baseline to follow-up stratified by educational level. Values are estimated marginal means from multivariable generalized linear mixed models. *P* values are for factor by time interaction terms. The error bars indicate 95%CIs.

A

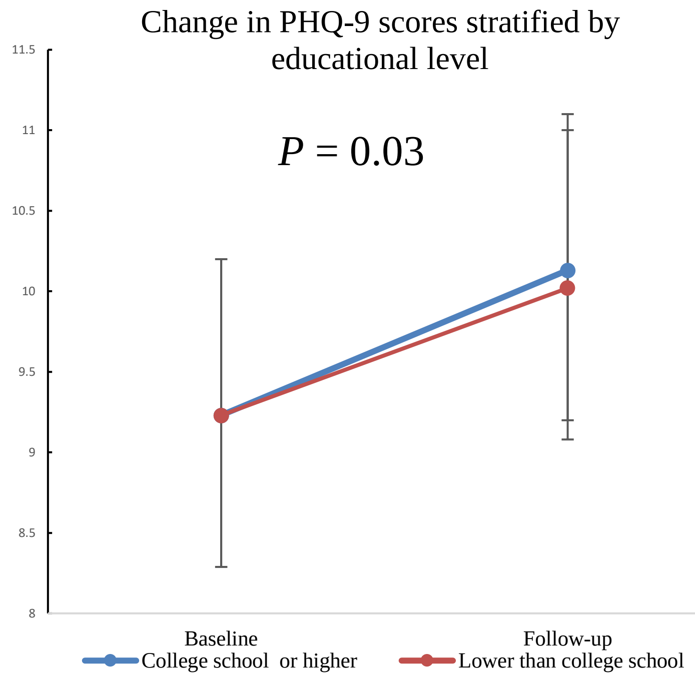

B

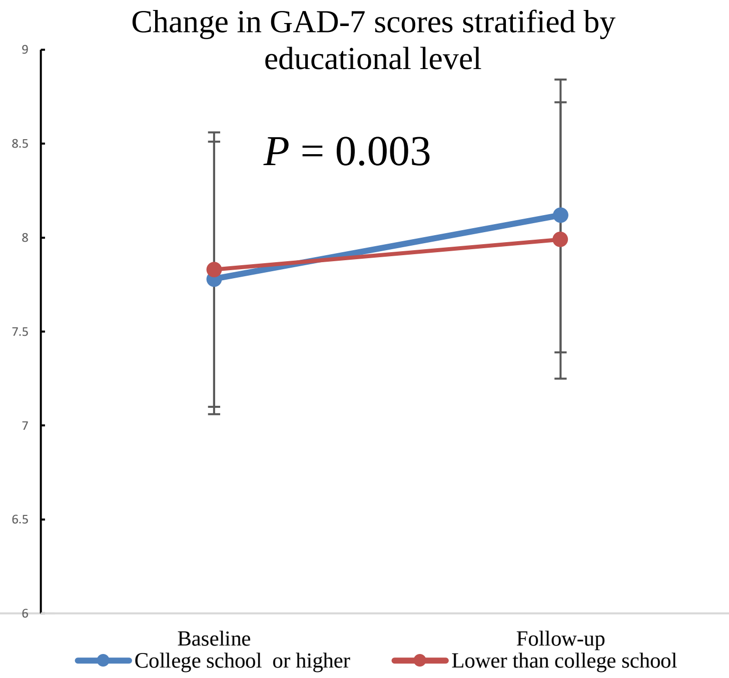

C

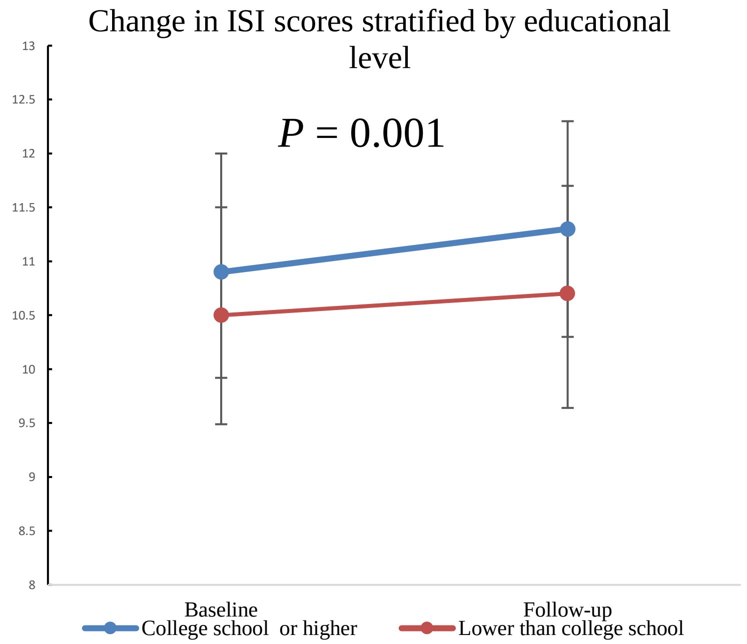

Figure S4- Changes in scores of depression, anxiety and insomnia from baseline to follow-up stratified by voluntarily wearing face masks. Values are estimated marginal means from multivariable generalized linear mixed models. *P* values are for factor by time interaction terms. The error bars indicate 95%CIs.

A

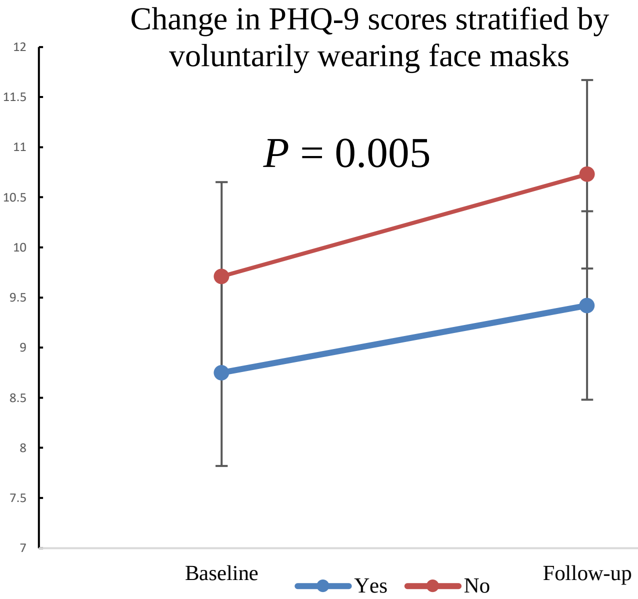

B

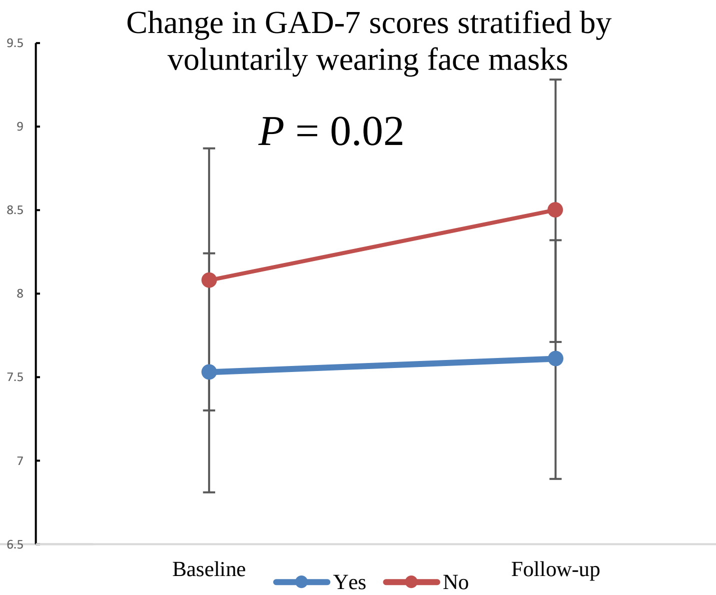

C

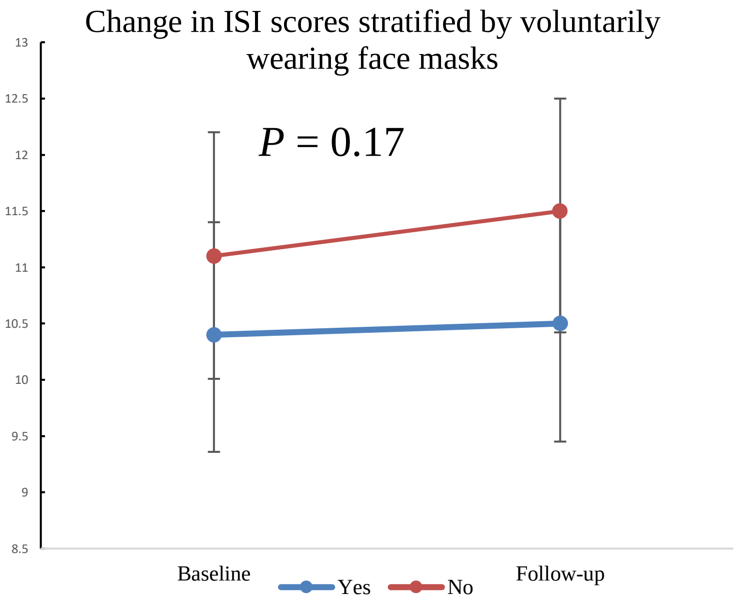

Figure S5- Changes in scores of depression, anxiety and insomnia from baseline to follow-up stratified by voluntarily reducing social gatherings. Values are estimated marginal means from multivariable generalized linear mixed models. *P* values are for factor by time interaction terms. The error bars indicate 95%CIs.

A

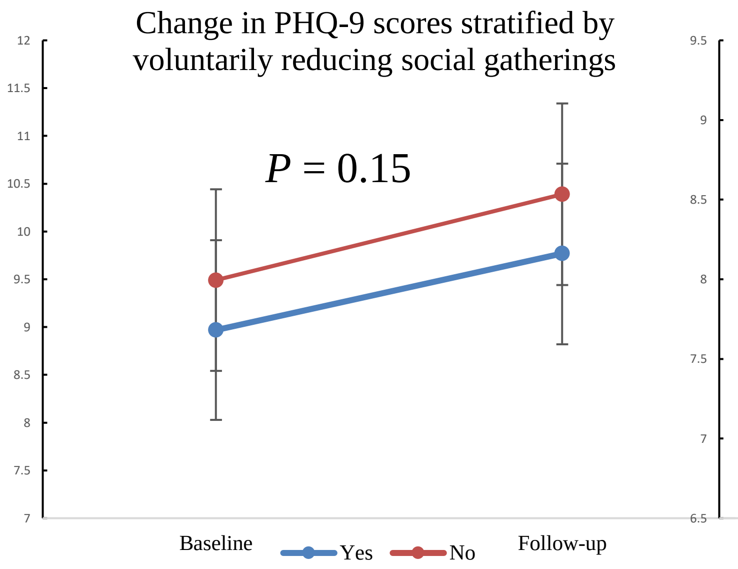

B

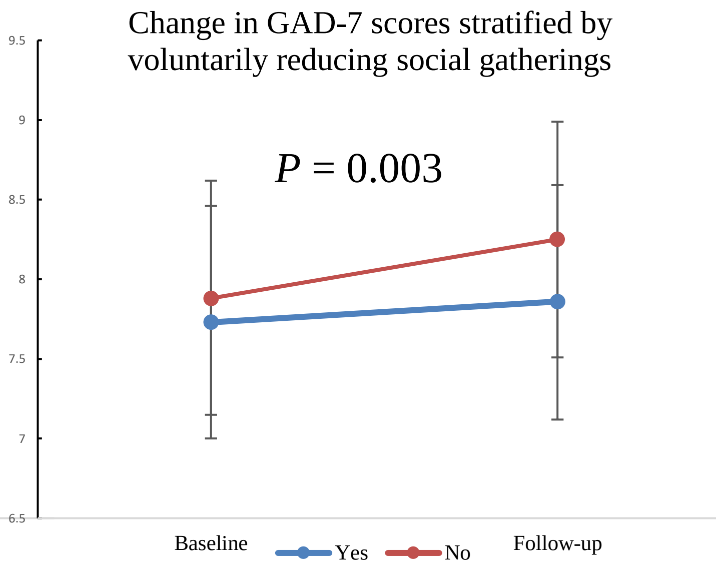

C

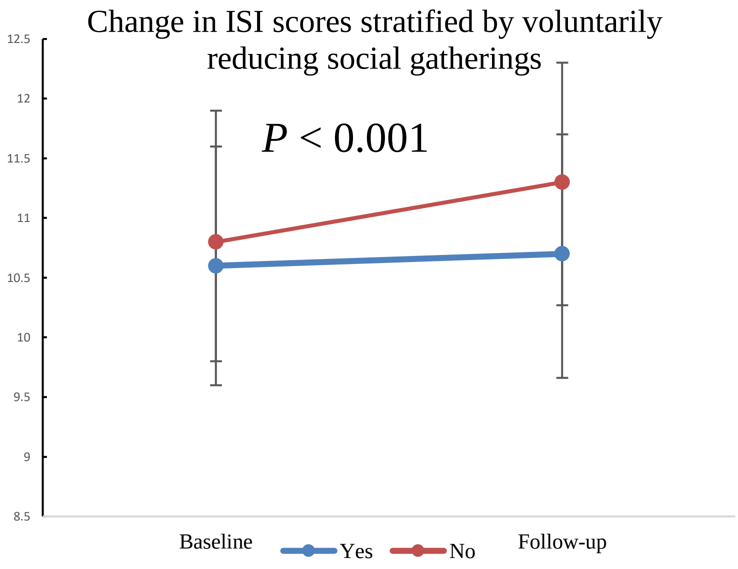

Figure S6- Changes in scores of depression, anxiety and insomnia from baseline to follow-up stratified by seeking psychological consultation. Values are estimated marginal means from multivariable generalized linear mixed models. *P* values are for factor by time interaction terms. The error bars indicate 95%CIs.

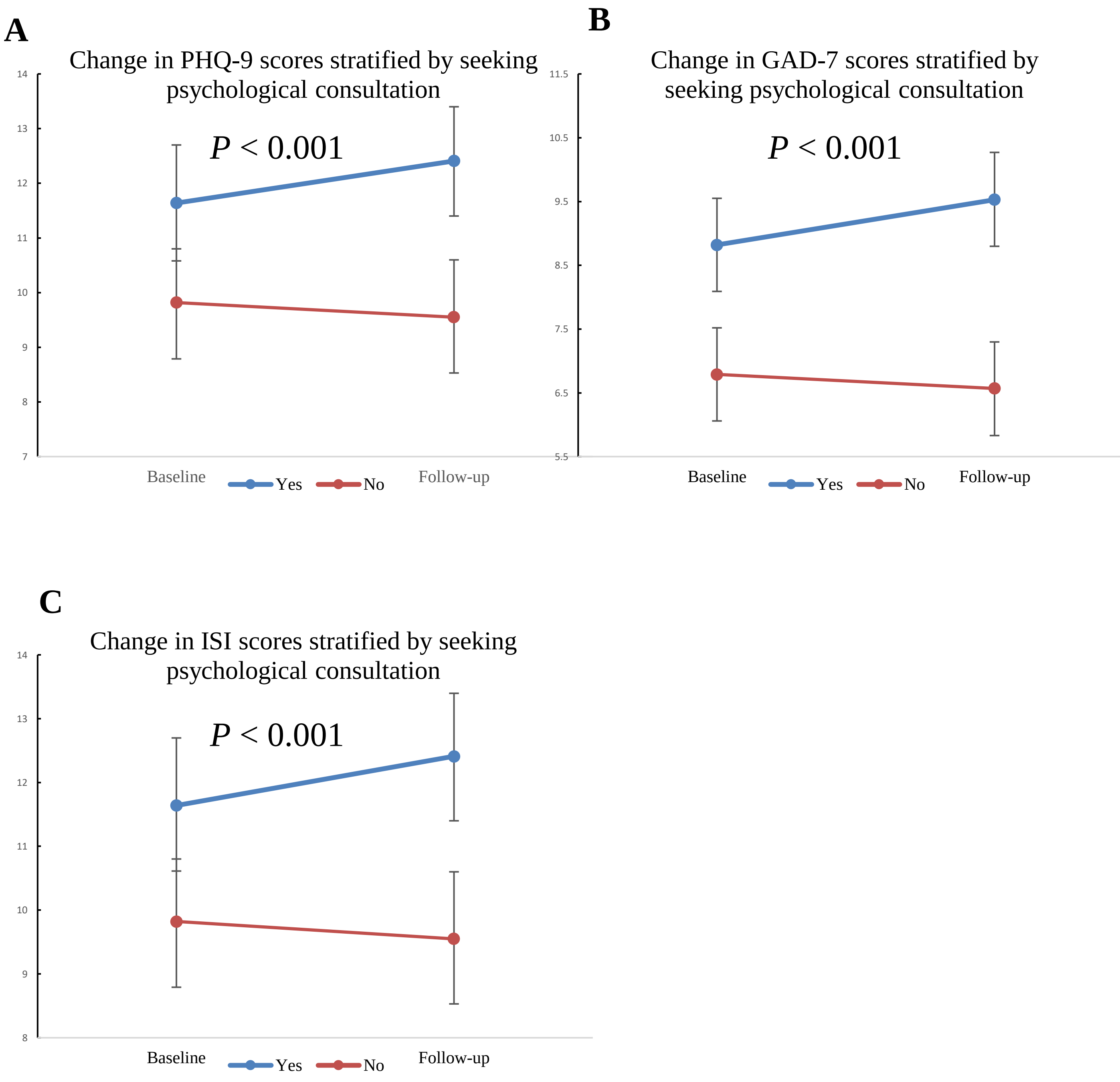

Supplement: Supplementary file 1 [file ijerph-18-08790-s001.zip › ijerph-1311619-supplementary.pdf]
